# Neurotoxic Effect of Benzo[a]pyrene and Its Possible Association with 6-Hydroxydopamine Induced Neurobehavioral Changes during Early Adolescence Period in Rats

**DOI:** 10.1155/2016/8606410

**Published:** 2016-02-29

**Authors:** Saroj Kumar Das, Bhupesh Patel, Manorama Patri

**Affiliations:** ^1^Department of Zoology, School of Life Sciences, Ravenshaw University, Cuttack, Odisha 753003, India; ^2^Department of High Altitude Physiology, Defence Institute of High Altitude Research, Leh 901205, India

## Abstract

Exposure to persistent genotoxicants like benzo[a]pyrene (B[a]P) during postnatal days causes neurobehavioral changes in animal models. However, neurotoxic potential of B[a]P and its association with 6-hydroxydopamine (6-OHDA) induced neurobehavioral changes are yet to be explored. The growth of rat brain peaks at the first week of birth and continues up to one month with the attainment of adolescence. Hence, the present study was conducted on male Wistar rats at postnatal day 5 (PND 5) following single intracisternal administration of B[a]P to compare with neurobehavioral and neurotransmitter changes induced by 6-OHDA at PND 30. Spontaneous motor activity was significantly increased by 6-OHDA showing similar trend following B[a]P administration. Total distance travelled in novel open field arena and elevated plus maze was significantly increased following B[a]P and 6-OHDA administration. Neurotransmitter estimation showed significant alleviation of dopamine in striatum following B[a]P and 6-OHDA administration. Histopathological studies of striatum by hematoxylin and eosin (H&E) staining revealed the neurodegenerative potential of B[a]P and 6-OHDA. Our results indicate that B[a]P-induced spontaneous motor hyperactivity in rats showed symptomatic similarities with 6-OHDA. In conclusion, early postnatal exposure to B[a]P in rats causing neurobehavioral changes may lead to serious neurodegenerative consequences during adolescence.

## 1. Introduction

Exposure to environmental contaminants poses a significant threat to normal growth and differentiation of the developing brain [1]. It has also been reported that nervous system is susceptible to toxic chemicals and subsequent developmental perturbation may lead to long-term irreversible consequences that affect nervous system function in adult animals [2]. Benzo[a]pyrene (B[a]P), a polycyclic aromatic hydrocarbon (PAH), is known for its neurotoxic potential causing neurobehavioral alterations in animal models [3]. Reports also suggest that exposure to B[a]P through early postnatal development leads to impairment in locomotor activity in adolescence [4]. A recent report revealed that subchronic oral administration of B[a]P in rats leads to spontaneous locomotor hyperactivity [5]. Behavioral and motor dysfunction following exposure to environmental toxicants like B[a]P is well documented but the neurodegenerative potential of these compounds cannot be ignored.

Exposure to B[a]P produces neuromuscular, physiological, autonomic abnormalities and also shows decreased responsiveness to sensory stimuli [6, 7]. Earlier reports suggest that microinjection of diesel exhaust fraction containing PAH into rat hippocampus and striatum leads to neuronal lesions [8]. Epidemiological study also revealed that parental occupational exposure to PAH was associated with an increased risk of neuroectodermal tumors in children [9]. Maternal exposure to airborne PAHs during pregnancy was associated with a reduction in head circumference, lower IQ, and reduced cognitive functioning in children [10].

Investigation into pathophysiological manifestations following exposure to persistent anthropogenic neurotoxicants like benzo[a]pyrene might address new insight into their neurodegenerative potential. Studies have also addressed the effect of highly specific neurotoxin 6-hydroxydopamine (6-OHDA) on catecholaminergic neurons with an extensive and irreversible loss of dopaminergic neurons in the mesencephalon which is associated with behavioral deficits [11–13]. Investigation of potential environmental neurotoxicants like B[a]P in animal model provides a new insight into serious human neurodegenerative diseases.

The purpose of the present study was to investigate the neurotoxic potential of B[a]P following intracisternal administration during early phase of postnatal development and its consequences in early adolescent period of rats. We also investigated the neurotoxic effect of benzo[a]pyrene and its possible association with 6-hydroxydopamine induced neurobehavioral changes during early adolescence period.

## 2. Materials and Methods

### 2.1. Chemicals and Reagents

The chemicals used in this experimentation were procured from Sigma-Aldrich Chemicals (St. Louis, MO, USA), unless otherwise mentioned such as benzo[a]pyrene (B[a]P, catalogue number B1760), corn oil (catalogue number C8267), 6-hydroxydomine hydrochloride (6-OHDA, catalogue number H4381), and paraformaldehyde.

### 2.2. Experimental Animals

Pregnant Wistar rats (*Rattus norvegicus*) were maintained in home cages in standard laboratory conditions and fed laboratory chow and filtered water ad libitum. The animals were subjected to 12 hr light and 12 hr dark cycle in standard laboratory environment. Temperature and humidity were maintained at 25–28°C and 60–65%, respectively.

### 2.3. Experimental Design and Toxicant Administration

Five-day-old male Wistar pups were arbitrarily consigned to lactating dams and intracisternal administration of B[a]P and 6-OHDA was carried out at postnatal day 5 [14]. The B[a]P solution was freshly prepared by dissolving 2 *μ*g/kg BW of B[a]P into 10 *μ*L of corn oil and was subsequently used for intracisternal administration while the control rats received only corn oil (10 *μ*L). Rats of 6-OHDA group were injected with 25 g/kg, desipramine in 100 *μ*L (i.p.), 30 minutes before 6-OHDA injection. Then 150 *μ*g/kg BW of 6-OHDA dissolved in 10 *μ*L of saline (containing 0.2% ascorbic acid as antioxidant) was injected into rats of 6-OHDA group while rats of control group received 10 *μ*L saline solution, respectively [15]. Five-day-old male Wistar pups were designated into four groups (12 male pups/group), namely, control A (corn oil); B[a]P (B[a]P dissolved in corn oil); control B (saline); and 6-OHDA (6-OHDA dissolved in saline). We aimed to study the neurodegenerative potential of B[a]P and its symptomatic similarities with 6-OHDA induced neurobehavioral alteration, rather than address the confounding effect as a result of coadministration of these neurotoxicants.

### 2.4. Evaluation of Spontaneous Motor Activity (SMA)

The standard experimental paradigm was followed for evaluation of SMA with slight modification [5]. SMA of rats at PND 30 was individually measured in a home cage with a Supermex system (Muromachi Kikai, Ikeda, Japan). The sensor monitored the motion and movement of the animal. Rats were maintained on a 12 h light: dark cycle during the recording and total distance covered for a period of 24 hr was recorded and the data was represented in 2 hr intervals as absolute time. Food and water were supplied at the beginning of recordings and rats were never disturbed in anyway.

### 2.5. Open Field Test

Open field test was conducted to assess the explorative behavior of rats [16]. Open field activity was monitored in a circular arena (75 m diameter and 1.5 cm height) wall with grey color paint. The animals were placed individually in a specific side of the wall of the arena and the time spent in the central zone and total distance travelled were automatically monitored for 5 min using ANY Maze software (Stoelting Co., USA). The apparatus was cleaned with 70% ethanol solution after testing each subject.

### 2.6. Elevated Plus Maze Test

Briefly, elevated plus maze consisted of two opposite open arms (50 cm long × 10 cm wide) and two enclosed arms (50 × 10 × 40 cm) that extended from a common central platform (10 × 10 cm), elevated 75 cm above the floor. Time spent in open arm and total distance travelled were recorded and considered as an indicator of general locomotor activity independent of anxiety [17]. The video-tracking system (ANY Maze software, Stoelting Co., USA) was set for 5 min of recording. The apparatus was cleaned with 70% ethanol and dried with paper towels before testing with another rodent.

### 2.7. Sample Preparation and Analysis

After completion of behavioral recording, rats of all the experimental groups were sacrificed after being deeply anesthetized with sodium pentobarbital and subsequently perfused intracardially with ice-cold 0.1 M phosphate buffer saline (PBS) followed by 4% paraformaldehyde [18]. Brain samples were cryoprotected in 30% sucrose in PBS for 48 hr and then serial cryosectioning was carried out for histopathological studies. For biochemical and molecular study, rats were sacrificed by cervical dislocation and the requisite brain region was immediately dissected out at 4°C in ice-cold 0.1 M PBS and then snap frozen in liquid nitrogen. The samples were then stored at −80°C until further analysis.

### 2.8. Quantitative Estimation of Dopamine by HPLC-ECD

Quantification of striatal dopamine level was carried out by high performance liquid chromatography-electrochemical detector (HPLC-ECD) with minor modification [19]. Briefly, 100 mg wet brain tissue (striatum) was homogenized by sonification in 0.5 mL of 0.2 M HClO_4_ (perchloric acid) containing isoproterenol as an internal standard substance. The homogenized tissue was then kept in ice bath for 30 min. and then subjected to centrifugation for 2 min. at 20,000 ×g and then the supernatant was added with 1 M sodium acetate to adjust the pH 3.0. After that 10 *μ*L of sample solution was injected into a separation column (Eicompak SC-5ODS, ID 3.0 × 100 mm) in a HPLC-ECD system for measurement of dopamine and the result was expressed as ng/mg of protein.

### 2.9. Histopathological Study by Hematoxylin and Eosin Staining

Brains were postfixed in 4% paraformaldehyde for 24 hr and then washed for 24 h under running tap water and dehydrated in graded series of ethanol concentrations (50%, 70%, 90%, and 100%) [20]. Then they were cleared in xylene, embedded in paraffin wax, serially sectioned at 7 mm thickness, and stained with hematoxylin and eosin (H&E). The slides were observed under microscope and images of striatal region were taken with requisite magnification with the help of a digital camera (Olympus, BX43F, Japan). The area and perimeter of the neurons were measured with the help of ocular micrometer.

### 2.10. Statistical Analysis

The mean and standard error of mean of each set, that is, control A, B[a]P, control B, and 6-OHDA, were calculated. The post hoc analysis was done by the Newman-Keuls test in all experimental groups by using one-way ANOVA. Difference below the probability level 0.05 was considered statistically significant. Bonferroni posttest was conducted to study the effect of B[a]P and 6-OHDA on SMA by using two-way ANOVA and difference below the probability level *p* < 0.001 was considered statistically significant.

## 3. Results

### 3.1. Assessment of Spontaneous Motor Activity

**Figure 1:**
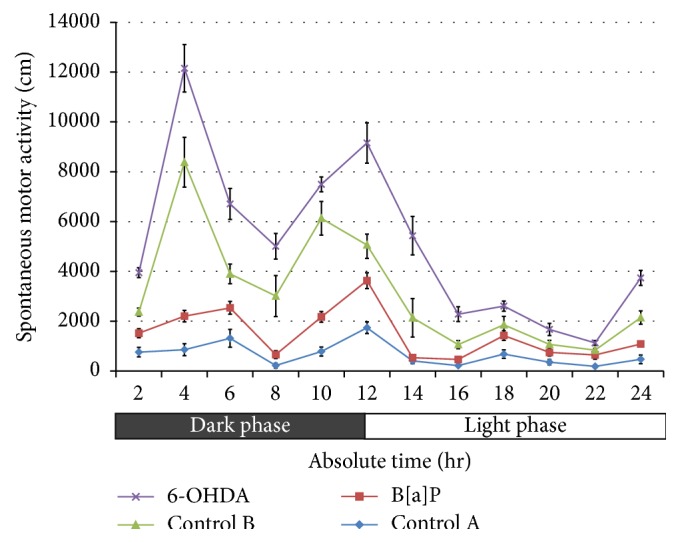
Spontaneous motor activity (SMA). Graph showing significant increase (*p* < 0.001) in SMA after 24 hrs of recording following B[a]P and 6-OHDA administration. The data were represented as mean ± SEM (*n* = 12/group).

B[a]P administration to rat neonates at PND 5 led to significant increase in spontaneous motor activity during 24 hr of recording (*F*_(3,528)_ = 380.8, *p* < 0.001) when compared to rats of control A group (Figure 1). Similarly, intracisternal administration of 6-OHDA to rats pups significantly increased the spontaneous motor activity (*F*_(3,528)_ = 380.8, *p* < 0.001) with respect to control B and B[a]P treated rats (Figure 1).

### 3.2. Open Field Test (OFT)

**Figure 2:**
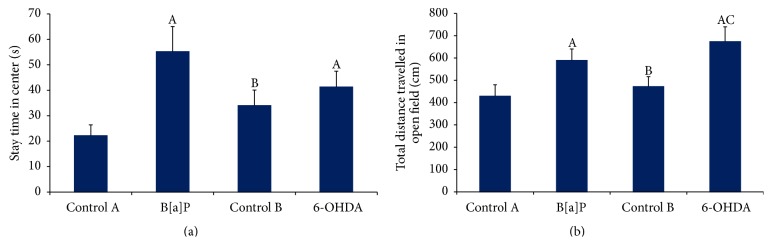
Open field test. Graphs showing alterations in time spent in central zone and total distance travelled in open field arena ((a) and (b)). Values are expressed as mean ± SEM (*n* = 12/group). “A” denotes *p* < 0.05 when compared to control A group, “B” denotes *p* < 0.05 when compared to B[a]P group, and “C” denotes *p* < 0.05 when compared to control B group.

Open field test showed significant increase in the stay time at center in B[a]P group as compared to control A (*F*_(3,44)_ = 11.71, *p* < 0.05) (Figure 2(a)). However the total distance travelled in OFT was significantly augmented following 6-OHDA administration (*F*_(3,44)_ = 14.69, *p* < 0.05) as compared to control B group (Figure 2(b)). Similarly, there was a significant increase in total distance travelled in B[a]P treated group when compared to control A (*F*_(3,44)_ = 14.69, *p* < 0.05). The results showed significant increase in total distance travelled in OFT following 6-OHDA administration (*F*_(3,44)_ = 14.69, *p* < 0.05) which showed similar effects following B[a]P administration.

### 3.3. Elevated Plus Maze Test (EPM)

**Figure 3:**
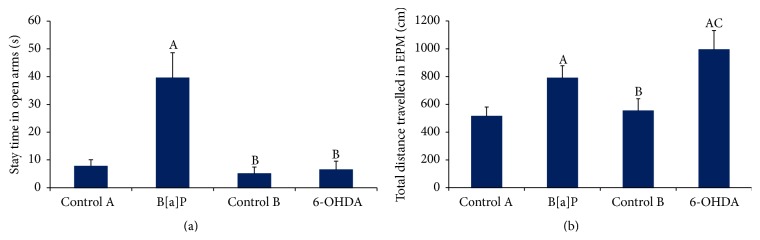
Elevated plus maze test. Graphs showing alterations in (a) time spent in open arm and (b) total distance travelled in elevated plus maze. Values are expressed as mean ± SEM (*n* = 12/group). “A” denotes *p* < 0.05 when compared to control A group, “B” denotes *p* < 0.05 when compared to B[a]P group, and “C” denotes *p* < 0.05 when compared to control B group.

The analysis of the stay time in open arm following B[a]P administration showed significant increase when compared to the control A group (*F*_(3,44)_ = 84.96, *p* < 0.05) whereas no significant difference was observed between 6-OHDA and control B groups (Figure 3(a)). The B[a]P and 6-OHDA administration also showed significant increase in total distance travelled in EPM (*F*_(3,44)_ = 28.23, *p* < 0.05) as compared to control A and control B, respectively (Figure 3(b)).

### 3.4. Measurement of Dopamine (DA) in Striatum

**Figure 4:**
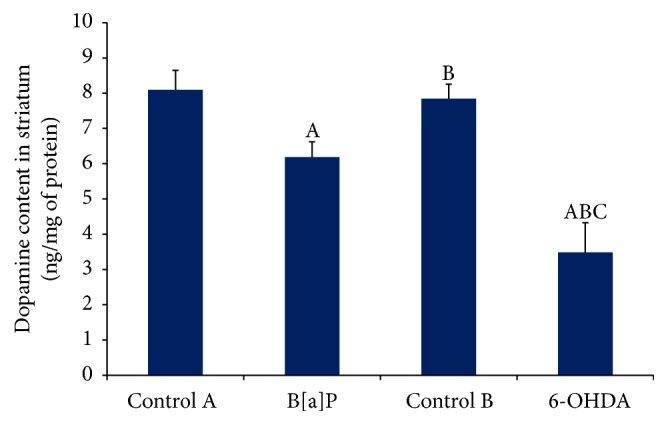
Quantification of dopamine level in striatum by HPLC-ECD. The graph showed significant alteration in striatal dopamine level following B[a]P and 6-OHDA administration when compared to their respective control groups. Values are expressed as mean ± SEM (*n* = 6/group). “A” denotes *p* < 0.05 when compared to control A group, “B” denotes *p* < 0.05 when compared to B[a]P group, and “C” denotes *p* < 0.05 when compared to control B group.

Dopamine (DA) level in striatum significantly (*F*_(3,20)_ = 14.79, *p* < 0.05) declined in B[a]P treated group as compared to control A (Figure 4). Comparable depletion of DA was detected in striatum following intracisternal administration of B[a]P as compared to 6-OHDA group (*F*_(3,20)_ = 14.79, *p* < 0.05) (Figure 4).

### 3.5. Histopathological Assessment of Striatum by Hematoxylin and Eosin (H&E) Staining

**Figure 5:**
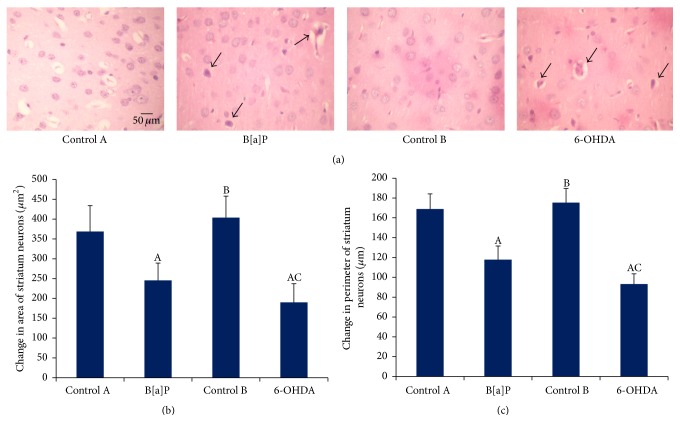
Histopathological study by hematoxylin and eosin staining. Representative microscopic images of hematoxylin and eosin staining of striatal neurons (a). Magnification at 10x (scale bar = 50 *μ*m). Arrow head depicts degenerative neurons. Histogram represents striatal neuronal area and perimeter ((b) and (c)). Values are expressed as mean ± SEM (*n* = 6/group). “A” denotes *p* < 0.05 when compared to control A group, “B” denotes *p* < 0.05 when compared to B[a]P group, and “C” denotes *p* < 0.05 when compared to control B group.

The histopathological examination of striatal region following H&E staining showed significant increase in abnormal striatal neuron morphology in B[a]P and 6-OHDA treated groups (Figure 5(a)). The mean area (*F*_(3,20)_ = 7.985, *p* < 0.05) and perimeter (*F*_(3,20)_ = 8.603, *p* < 0.05) of striatal neurons were significantly decreased in B[a]P treated group as compared to control A and similar alleviation of mean area (*F*_(3,20)_ = 7.985, *p* < 0.05) and perimeter (*F*_(3,20)_ = 8.603, *p* < 0.05) of striatal neurons was also observed in 6-OHDA group as compared to control B (Figures 5(b) and 5(c)). However, there was a significant increase in pyknotic cell counts in 6-OHDA group when compared with B[a]P.

## 4. Discussion

Polycyclic aromatic hydrocarbons (PAHs) are predominantly anthropogenic in source and are liberated as a consequence of partial combustion carbon containing organic compounds [21, 22]. Benzo[a]pyrene (B[a]P) is extensively used as a prototype of PAH which demonstrates significant genotoxicity. Further, humans and rodents differ with respect to their sensitivity to varying doses of B[a]P and previous findings suggested that humans were 10–100 times more sensitive to toxic effects of B[a]P than rodents [23]. Additionally, infants and children are exceptionally susceptible to environmental neurotoxicants at levels far below those known to harm adults [24]. As the early phase of postnatal life is involved with rapid phase of growth and development, it becomes a target of exposure to environment neurotoxicants, the rapid growth period in rat neonates straddling the first (1-2) weeks of life to acquire numerous novel motor and sensory skills during the early stage of development [25].

B[a]P induced motor dysfunction leading to spontaneous motor hyperactivity in rodents addresses possible consequences of serious neurological problems [5]. Several studies have been conducted to show spontaneous motor dysfunction and its association with neurodegenerative symptoms following administration of potent neurotoxicants [26, 27]. Substantial evidence suggests that early postnatal exposure to B[a]P drastically alters the motor functions [28]. This neurodegenerative predisposition is potentially related to alleviated dopamine level at striatum [29]. During early stage of postnatal development striatal dopaminergic neurons regulate the storage and release of dopamine. As the blood-brain barrier is not fully developed in rat neonates, 6-OHDA, a potent neurotoxicant, might adversely affect the brain development during early adolescence period.

Hence, the present study was conducted at PND 5 in male Wistar pups following single intracisternal administration of B[a]P that showed significant increase in spontaneous motor activity at PND 30. The above findings are in agreement with the earlier studies [5]. Further investigation showed that neonatal 6-OHDA administration leads to spontaneous motor hyperactivity in adolescent rats and the findings are in agreement with previous reports [30]. Novel open field exploration study was also considered to address the spontaneous locomotor activity [31]. Our results demonstrated that 6-OHDA administration leads to significant increase in total distance travelled in both OFT and EPM and similar behavioral change was also found after B[a]P administration. The motor dysfunction following B[a]P administration was found to be positively associated with the neurobehavioral manifestation induced by potent neurotoxicant, that is, 6-OHDA, and the above findings are in agreement with previous reports [32, 33]. The behavioral observations as stay time in OFT and EPM significantly increased in the group to which B[a]P was administered as compared to control. However, no significant difference was observed after 6-OHDA administration, from which it can be inferred that intracisternal B[a]P administration has anxiolytic potential and the findings are in agreement with the previous report [4].

We extended our findings to ascertain the possible relationship of neurobehavioral manifestation induced by intracisternal administration of B[a]P with that of dopamine level in striatum of adolescent rats. Our results showed significant alleviation of striatal dopamine level following B[a]P administration and the result finds support from previous report [20]. Similarly, 6-OHDA administration leads to significant decrease in dopamine level as compared to B[a]P and the above findings are in agreement with previous report [34, 35]. The possible effect of neonatal B[a]P administration on striatal dopamine level during adolescence was found to be linked with the effect of 6-OHDA. The findings of striatal dopamine alleviation by B[a]P administration were significantly associated with altered histopathological observation. Our results demonstrated a significant decrease in area and perimeter of striatal neurons following B[a]P administration. The above findings are comparable with the neurodegenerative symptoms exhibited by 6-OHDA and our results are supported by previous report [15]. The possible neurodegenerative consequences following B[a]P administration at PND 5 and its neurobehavioral manifestation in adolescent rats may be associated with reduced striatal dopamine level. The basic findings of the present study indicate that 6-OHDA produces alterations in behavior that are similar to those produced by B[a]P.

## 5. Conclusion

In conclusion, the findings suggest that early postnatal life is highly susceptible to environmental neurotoxicants leading to neurobehavioral perturbation in adolescence. Early exposure to potent environmental neurotoxicants like B[a]P may lead to reduction in dopamine neurotransmitter level that can cause striatal neurodegeneration. We addressed a pioneer effect of B[a]P exposure during early postnatal life leading to neurodegenerative processes and its symptomatic similarities with progressive neurodegenerative diseases.
